# Supplementary material for: The Effects of Selenium on Wheat Fusarium Head Blight and DON Accumulation Were Selenium Compound-Dependent
Source: Toxins (Basel). 2020 Sep 6;12(9):573. doi: 10.3390/toxins12090573 (PMC7551897; doi:10.3390/toxins12090573)
Supplement: Supplementary file 1 [file toxins-12-00573-s001.pdf]

Supplementary Materials: The Effects of Selenium on Wheat Fusarium Head Blight and DON Accumulation were Selenium Compound-Dependent

Xueyun Mao, Chen Hua, Liang Yang, Yuhui Zhang, Zhengxi Sun, Lei Li and Tao Li *

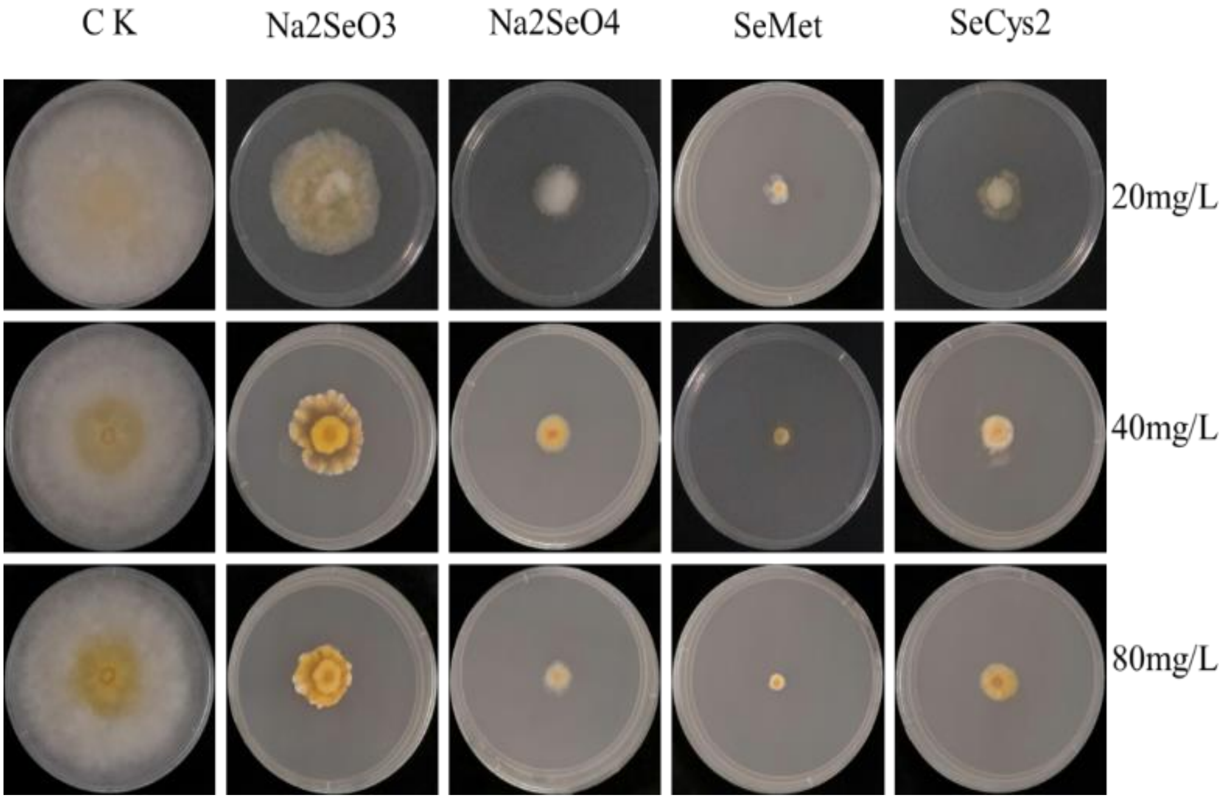

Figure S1. The mycelial colony cultured for 96 hours treated by different Se species.

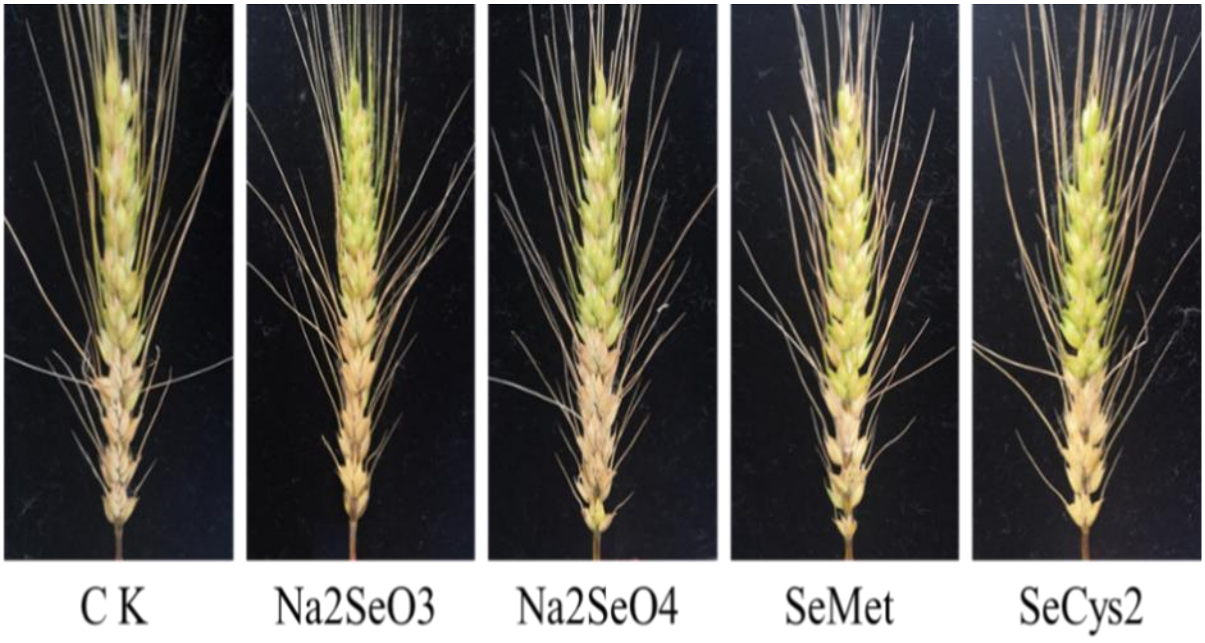

Figure S2. FHB severities at 25 days after inoculation under treatments with different Se species.

Table S1. The correlation analysis among PSS, DON content and Se content.

Trait		PSS	DON content	Se content
PSS	Pearson correlation	1		
	Significance (bilateral)			
DON content	Pearson correlation	0.757	1	
	Significance (bilateral)	0.139		
Se content	Pearson correlation	0.273	0.079	1
	Significance (bilateral)	0.657	0.899	

Technical Note: The Conditions of Chromatography and Mass Spectrometry Parameters

LC operating conditions:

1. Chromatographic column: Waters ACQUITY UPLC HSS T3, 2.1 ×1 00mm Column, 1.8 μm.

2. Mobile phase: aqueous phase (A), water + 0.1% formic acid; organic phase (B), methanol.

3. Gradient elution: 10% B (0.0 min–0.5 min), 35% B (0.5 min–1.0 min), 50% B (1.0 min–1.5 min), 65% B (1.5 min–3.0 min), 90% B (3.0 min–5.0 min), 10% B (5.0 min–8.0 min).

4. Flow rate of mobile phase: 0.3 mL/min.

5. Column temperature: 40 °C.

6. Sample size: 10 μL.

Mass spectrometry parameters:.

Name	Parent (m/z)	Product (m/z)	SRM Collision Energy (eV)	Retention Time (min)	Time Window (min)	Polarity
DON	297.1	91.1	40	5.00	8.00	+
DON	297.1	175.2	15	5.00	8.00	+

Limit of determination is 1 μg/kg.; +, positive ion mode
